# Whole‐genome sequencing of 1,083 HPV45 cases and controls identifies genetic variants associated with glandular cervical lesions

**DOI:** 10.1002/ijc.35464

**Published:** 2025-05-05

**Authors:** Aimee J. Koestler, Chase W. Nelson, Meredith Yeager, Zigui Chen, Sambit K. Mishra, Laurie Burdett, Michael Dean, Elizabeth Suh‐Burgmann, Thomas Lorey, Gary M. Clifford, Nicolas Wentzensen, Philip E. Castle, Mark Schiffman, Robert D. Burk, Lisa Mirabello

**Affiliations:** ^1^ Division of Cancer Epidemiology and Genetics National Cancer Institute (NCI), NIH Rockville Maryland USA; ^2^ Cancer Genomics Research Laboratory Frederick National Laboratory for Cancer Research Frederick Maryland USA; ^3^ Department of Biology Hood College Frederick Maryland USA; ^4^ Department of Microbiology The Chinese University of Hong Kong Hong Kong China; ^5^ Division of Research Kaiser Permanente Northern California (KPNC) Oakland California USA; ^6^ Regional Laboratory and Women's Health Research Institute, Division of Research KPNC Oakland California USA; ^7^ Early Detection, Prevention and Infections Branch International Agency for Research on Cancer (IARC) Lyon France; ^8^ Division of Cancer Prevention NCI, NIH Bethesda Maryland USA; ^9^ Department of Pediatrics, Epidemiology and Population Health, Microbiology and Immunology, and Obstetrics & Gynecology and Women's Health Albert Einstein College of Medicine New York City New York USA

**Keywords:** cervical adenocarcinoma, cervical carcinogenesis, HPV genomics, HPV variants, HPV45

## Abstract

Human papillomavirus type 45 (HPV45) causes ~6% of all cervical cancers and an even greater proportion of adenocarcinomas, the latter of which are challenging to detect using current cervical cancer screening. Little is known about how HPV45 genetic variation is related to the risk of cervical precancer/cancer. To investigate this, we whole‐genome sequenced a total of 1,083 HPV45‐positive samples from two large studies. We evaluated associations of HPV45 genetic variation (sublineages, subclades, and SNPs) with histology‐specific precancer/cancer risk using logistic regression and evaluated risk modification by self‐reported race/ethnicity. Compared to the common A1 sublineage, A2 and B1 were associated with increased precancer/cancer (A2, OR = 3.9, 95% CI = 1.9–8.5; B1, OR = 2.7, 95% CI = 1.3–5.8; B2, OR = 3.3, 95% CI = 1.6–7.3), and most strongly with the glandular precancers/cancers (AIS/ADC; A2, OR = 6.9, 95% CI = 1.0–184; B1, OR = 6.2, 95% CI = 1.1–159). The A2 sublineage was most prevalent in women in East Asia and women who self‐reported as Asian/Pacific Islander (PI) in the U.S.; East Asian and Asian/PI women had the greatest precancer/cancer risk associated with A2 infections (OR = 5.8, 95% CI = 1.3–37.4) compared to all other sublineages among these women. We further evaluated precancer/cancer risk associations for 262 individual HPV45 SNPs and identified four SNPs significantly associated with only glandular precancers/cancers after correction for multiple tests (ORs ranged 7.8–20.7). One of these SNPs was a nonsynonymous variant in both overlapping viral E2/E4 ORFs. In summary, we show that HPV45 genetic variation influences the risk of precancer/cancer, specifically glandular precancer/cancer. Further studies of these genetic variants may improve our understanding of glandular lesions.

## Graphical abstract

What's new?

Little is known about how genetic variation in human papillomavirus type 45 (HPV45) influences its ability to cause cancer. Here, the authors conducted the largest whole‐genome sequencing study to date of HPV45, looking for associations between viral genetic variation and precancer or cancer risk. From 1,083 samples, they found that sublineages A2 and B1 were associated with increased precancer/cancer risk specific to certain histologies. They also identified four single nucleotide polymorphisms (SNPs) associated solely with glandular precancer/cancer.

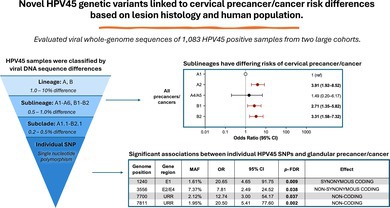

## 1 INTRODUCTION

Cervical cancer is the fourth most commonly diagnosed cancer and cause of death in women worldwide; over 600,000 new cervical cancer cases and 340,000 deaths were estimated in 2020, particularly in low‐ and middle‐income countries.^1^ In the U.S., cervical cancer screening has led to a decline in cervical cancer incidence and mortality due to the detection and treatment of squamous cell carcinoma (SCC) and its precursors.^2^, ^3^ However, cervical adenocarcinoma (ADC), the less common histologic subtype, is less effectively detected with current screening programs. This is attributed to the development of glandular lesions in the endocervical canal, which can fail to be sampled by cytology.^3^ Consequently, rates of ADC are rising in the U.S. and other developed countries.^3^, ^4^, ^5^

HPVs are classified based on their genetic composition into genera, species, and types.^6^ Persistent infection with one of the approximately 13 high‐risk HPV (HR‐HPV) types causes virtually all cervical cancer cases, and the prevalence of these HR‐HPV types varies between geographic regions.^6^ HPV16 is the most prevalent and carcinogenic HR‐HPV type, followed by HPV18 and HPV45.^2^, ^3^, ^7^ Both HPV18 and HPV45 are disproportionately associated with a larger number of ADC cases compared to SCCs (HPV45: 8.5% of ADC vs. 4.3% of SCC), which is in contrast to HPV16, which causes about half of both ADC and SCC.^3^ The HR‐HPV types can be phylogenetically grouped based on their genetic similarities; HPV16 is a member of the *Alpha‐9* species, while HPV18 and HPV45 are members of the *Alpha‐7* species and are more closely genetically related.^8^

HR‐HPV types can be further divided into lineages (e.g., A, B) and sublineages (e.g., A1, A2) based on viral DNA sequence differences, defined by 1.0%–10.0% and 0.5%–1.0% nucleotide differences across the genome, respectively.^5^ This within‐type HPV genome variation (lineages, sublineages, and viral single nucleotide polymorphisms [SNPs]) has been associated with differences in cervical precancer/cancer risk.^5^, ^9^ We and others have shown that evolutionarily derived lineages and sublineages,^8^, ^10^, ^11^, ^12^, ^13^, ^14^, ^15^, ^16^, ^17^ and individual viral SNPs,^11^, ^18^, ^19^ are linked to differences in precancer and cancer risk for several HR‐HPV types.^9^ The geographic distribution of these sublineages is also important to consider because certain sublineages are enriched in specific populations and can be linked to variable precancer and cancer risk in such groups.^12^, ^13^ For example, HPV35 has a higher prevalence in women of African ancestry compared to women with a different ancestral background^11^, ^20^; its A2 sublineage and specific SNPs are associated with an increased precancer/cancer risk only in women of African ancestry.^11^

In a previous study using targeted HPV45 E6/E7 open reading frame (ORF) sequencing, we identified two HPV45 lineages composed of five distinctive sublineages (A1, A2, A3, B1, B2) that were variably distributed around the world, potentially reflecting coevolution with specific human populations.^8^ The HPV45 B2 sublineage was associated with more cervical cancer than the non‐B2 sublineages; this association varied by geographic region and was strongest in the samples from African women.^8^ However, another study did not identify significant differences between the HPV45 A and B lineages related to infection outcomes or persistence in a Costa Rican population.^13^ Further investigation is needed to understand HPV45 genetic variation and its role in precancer/cancer risk. We conducted a large case–control study to evaluate cervical precancer and cancer risk associated with HPV45 sublineages and viral SNPs using whole‐genome sequencing (WGS) and whether risk is modified by ethnicity.

## 2 MATERIALS AND METHODS

### 2.1 Study population

This study includes 774 samples (557 controls and 123 CIN2 and 94 CIN3/AIS/cancer cases) from the National Cancer Institute (NCI)‐Kaiser Permanente Northern California (KPNC) HPV Persistence and Progression (PaP) cohort and 309 samples (144 non‐cancer and 165 cancer cases) collected by the International Agency for Research on Cancer (IARC).

#### 2.1.1 PaP study

We designed a nested case–control study of HPV45 samples from the PaP cohort. This cohort has been previously described^21^ and includes 45,302 HPV‐positive women in the U.S. that underwent routine cervical cancer screening between December 2007 and January 2011 using cytology (first specimen) and HPV (second specimen). Cervical exfoliated cells were tested clinically using Hybrid Capture 2 (HC2; Qiagen Inc., Gaithersburg, MD, USA), which can detect the 13 pooled carcinogenic HPV types. Typing of archived specimens in neutralized specimen transport medium (STM; Qiagen Inc., Gaithersburg, MD, USA) was performed using a variety of assays, including Onclarity (BD, Franklin Lakes, NJ, USA), Linear Array (Roche Diagnostics, Indianapolis, IN, USA), or MY09‐MY11 PCR based on prior sub‐studies.^22^, ^23^

Infection outcomes were categorized based on the worst histology throughout the study follow‐up period (from 2007 to 2019). Cases were defined as women who were HPV45‐positive with cervical intraepithelial neoplasia (CIN) grade 2 or higher (CIN2+): 123 CIN2, 69 CIN grade 3 (CIN3), 14 adenocarcinoma in situ (AIS), 5 ADC, 5 SCC, and 1 cancer with unknown histology (Table S1). Controls were defined as women with baseline HPV45‐positive specimens that subsequently cleared their infection and/or had a benign infection defined as ≤CIN1 that did not progress to CIN2+ throughout the study. There were 103 HPV45‐positive samples that had co‐infections with HPV16 and/or HPV18: 73 with HPV16 only, 23 with HPV18 only, and 7 with both HPV16 and 18 (Table S2).

Clinical and demographic information was obtained from electronic medical records. Women self‐reported their race and ethnicity on their patient demographic forms as White, Asian or Pacific Islander (PI), Black, Hispanic, multiracial/other, or not reported.

#### 2.1.2 IARC samples

Samples were collected from 34 countries to assess the geographic distribution of HPV45 genetic variation since the number of controls and cancers were not equally collected from each country (Table S2). Of the 309 IARC samples included in our study, 300 were previously evaluated using targeted sequencing of the E6 and E7 ORFs in our previous study of HPV45.^8^ HPV detection and typing were performed using a GP5+/6+‐based PCR system on cervical cytology samples and frozen or formalin‐fixed paraffin‐embedded (FFPE) samples. In total, there were 165 HPV45‐positive cervical cancers and 144 HPV45‐positive non‐cancers (including 135 controls of normal, atypical squamous or glandular cells of undetermined significance [ASCUS], or low‐grade intraepithelial lesion [LSIL] cytology specimens, and 9 high‐grade squamous intraepithelial lesion [HSIL] or CIN2/CIN3) specimens (Table S2).

### 2.2 DNA extraction, library development, and next‐generation sequencing

DNA was extracted from each sample and whole‐genome sequenced using custom HPV45 Ion Torrent AmpliSeq panels (43 primer pairs) targeting the whole HPV45 genome. DNA underwent a library construction protocol according to the manufacturer's recommendation, using the AmpliSeq Library Preparation Kit 2.0‐96LV (Thermo Fisher Scientifics, Waltham, MA, USA) and custom oligonucleotide primers, designed by Life Tech in conjunction with lab personnel, that amplify 43 overlapping amplicons covering 100% of the HPV45 viral genome. The primers were pooled into two separate PCR reactions to avoid cross‐primer dimerization, and amplification was performed using Phusion High‐Fidelity DNA Polymerase (Thermo Fisher Scientifics, Waltham, MA, USA), with an error rate less than 1%. Individual libraries were quantified prior to sequencing using the Kapa Biosystems Library Quantification Kit‐IonTorrent/LightCycler 480 (Roche, Basel, Switzerland), and library concentration was determined using the Agilent BioAnalyzer DNA High‐Sensitivity LabChip (Agilent Technologies, Santa Clara, California). Average amplicon size was 195 bp. Up to 96 samples were pooled on Ion 540 chips for high‐throughput sequencing on a Thermo Fisher Life Science Ion Torrent S5 GeneStudio system (Thermo Fisher Scientifics, Waltham, MA, USA). Raw sequencing reads were quality assessed, trimmed, and mapped to the HPV45 reference sequence (GenBank accession X74479.1) using Ion Torrent Suite software (Thermo Fisher Scientifics, Waltham, MA, USA) and the Torrent Mapping Alignment Program (https://github.com/iontorrent/TS/tree/master/Analysis/TMAP). An in‐house pipeline was used for variant calling and variant annotation using Torrent Variant Caller v.5.0.3 and snpEff v.3.6c.^24^

Phylogenetic trees were created with HPV45 sublineage reference and consensus genome sequences for each sample using RAxML‐NG version 1.0.0.^25^ Sublineages were assigned using a custom clade assignment algorithm that averaged the sublineage assignments across a ‘plausible tree set’ of 200 maximum likelihood trees.^26^ For each tree, clade membership for each sequence was assessed using a bootstrap process in which the tree was randomly rooted and pruned 1000 times to identify monophyly with one of the eight sublineage reference genomes. Samples were then classified into one of eight sublineages (A1–6, B1–2). We excluded 86 PaP samples due to poor or inconsistent coverage across the viral genome, coinfection with multiple HPV45 sublineages where the predominant sublineage could not be identified, or limited bootstrap support for sublineage assignment. 31 IARC samples were excluded due to these same criteria. The sequencing coverage and quality statistics for each sample are summarized in Table S3.

**FIGURE 1:**
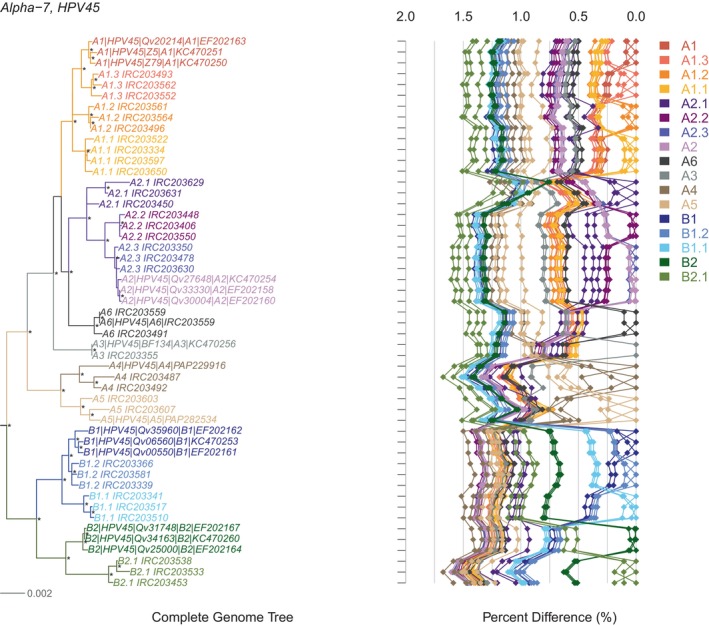
HPV45 phylogenetic tree of sublineages and subclades with pairwise distance comparisons using viral complete genomes. Pairwise comparisons of representative HPV45 sublineages (A1–A6, B1–B2) and subclades (e.g., A1.1, A1.2, and A1.3) genome sequences. The maximum likelihood (ML) tree was constructed using RAxML version 8.2.12, with bootstrap values ≥80% indicated with an asterisk (*) near specific branches. The percent nucleotide differences for each sublineage and subclade comparison are illustrated by the colored lines (shown in the panel to the right of the phylogeny). Values for each comparison are connected by lines, and the comparison to self is indicated by the 0.0% difference point.

In this study, we further defined a new taxonomic level of classification to highlight the distinct clades or evolutionary groups observed within sublineages: subclades. In our study, these subclades exhibited pairwise viral genome differences of 0.2%–0.5% from each other (Figure 1). Subclades were named based on the sublineage they formed a clade and an incremental decimal number (based on the order of discovery), as A1.1, A1.2, Al.3, A2.1, A2.2, A2.3, B1.1, B1.2, and B2.1, similar to nomenclature used for other viruses.^27^ More specifically, each decimal point implies “derived from/within” (e.g., A1.1 and A1.2 are both derived from A1).

### 2.3 Statistical analyses

Differences in the HPV45 sublineage distribution by infection outcomes, ethnicity, age group, and geographic region were evaluated by chi‐squared and Fisher's exact tests. Associations of HPV45 sublineages and individual SNPs with CIN2+/CIN3+ (cases), compared to controls (referent group), were assessed using logistic regression to calculate odds ratios (ORs) and 95% confidence intervals (95% CIs). We also evaluated all associations separately for glandular cases (AIS/ADC) and squamous cases (CIN3/SCC) compared to controls. For the sublineage analyses, we used the common A1 sublineage as the referent group. Analyses were performed with all HPV45‐positive samples and after excluding those with an HPV16 and/or HPV18 co‐infection. We also conducted association analyses adjusting for ethnicity; ethnicity was not associated with and did not impact the strength of the outcome associations; therefore, we are presenting the unadjusted model. For the individual SNP analyses, the most common nucleotide at each genomic position was used as the referent group; therefore, the tested minor allele was the less common allele based on the data and not based on the reference sequence. Significant *p‐*values were corrected for multiple comparisons using false discovery rate (FDR) based on the number of polymorphic sites with a minor allele frequency (MAF) ≥1%.

We then stratified and evaluated if there was effect modification of ethnicity on the associations between sublineages and CIN3+ status. For each ethnicity stratum, we assessed the association of each sublineage with CIN3+ (we did not stratify by lesion histology due to small counts) compared to women with all other sublineages combined of that ethnicity as the reference group. For the self‐reported ethnicity analyses, we excluded women who did not self‐report their ethnicity (*n* = 61) and women reporting multiple/other ethnicities (*n* = 9) in the PaP cohort (Table S2).

Aggregate testing was performed to evaluate the cumulative effects of multiple genetic variants in each viral gene region of HPV45 and the URR, stratifying by histologic outcome, using a previously developed customized “burden” test.^18^, ^28^ We evaluated the cumulative effects for all rare genetic variants and specifically rare non‐silent (i.e., nonsynonymous and nonsense) variants by each viral region in cases vs. controls. Rare variants included nucleotide variants that had an MAF of <1%. For the non‐coding URR, all rare variants were included in the burden test.

We used AlphaFold2^29^ to evaluate the 3D structure of the HPV45 E2 protein and DynaMut2^30^ to predict the thermodynamic stability changes with specific E2 mutations. For all analyses, when the sample size was less than five, Fisher's exact test was used for *p‐*value, OR, and 95% CI estimations. Statistical analyses were performed with R version 2022.12.0+353. All statistical tests were two‐sided, and FDR correction was performed to obtain the adjusted *p*‐value to account for multiple testing.

## 3 RESULTS

### 3.1 Distribution of HPV45 sublineages in PaP and IARC

**FIGURE 2:**
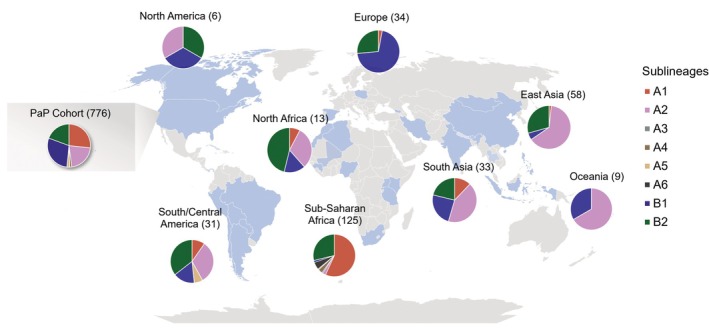
HPV45 sublineage distribution by geographic regions using 309 samples collected by IARC from 34 countries. Light blue highlighted countries represent the sites where samples were collected. Sublineage color legend identifies the corresponding color for each sublineage to illustrate the sublineage distribution in each pie chart for the main geographic regions. The distribution of sublineages in the NCI‐KPNC PaP cohort is shown as an inset pie chart to show the distribution in the U.S.

A total of 774 case–control samples from the PaP cohort were used to evaluate associations between HPV45 genetic variants and carcinogenesis, whereas the 309 IARC samples collected around the world were used to evaluate the geographic distribution of HPV45 sublineages (Table S4). The distribution of HPV45 sublineages varied by infection outcome in PaP (*p* = 0.011), self‐reported ethnicity (*p* = 4.998E‐04), infection outcome in IARC (*p* = 0.132), and geographic region (*p* = 4.998E‐04) (Table 1 and Table S4). In the IARC study, the B1 sublineage was prevalent in women from Europe, A2 was most enriched in women from East Asia, and A1 was most prevalent in women from Sub‐Saharan Africa (Figure 2 and Figure S2 [cancer cases only]). In the U.S. PaP cohort, nearly half of the women self‐reported as White (48.1%; Table S2), and the A1 and B1 sublineages were most common in White women (Table 1 and Table S4). The most prevalent sublineage varied by self‐reported ethnicity; among the Black women, the A1 sublineage was most common, and among the Asian/PI women, A2 was most prevalent (Table S4).

**TABLE 1:** Characteristics of HPV45 sublineages in 774 samples from the PaP cohort and 309 samples from the IARC study.

	Sublineage	A1		A2		A4		A5		A6		B1		B2		Total	
		*n*	col %	*n*	col %	*n*	col %	*n*	col %	*n*	col %	*n*	col %	*n*	col %	*n*	%
PaP	**Infection outcome**	**207**		**164**		**12**		**16**		**0**		**225**		**150**		**774**	
	Control	163	78.7	106	64.6	9	75.0	12	75.0	0	0.0	164	72.9	103	68.7	557	72.0
	CIN2	33	15.9	30	18.3	3	25.0	2	12.5	0	0.0	31	13.8	24	16.0	123	15.9
	CIN3/SCC	10	4.8	22	13.4	0	0.0	0	0.0	0	0.0	23	10.2	19	12.7	74	9.6
	AIS/ADC	1	0.5	5	3.0	0	0.0	2	12.5	0	0.0	7	3.1	4	2.7	19	2.5
	Cancer	0	0.0	1	0.6	0	0.0	0	0.0	0	0.0	0	0.0	0	0.0	1	0.1
	**Self‐reported ethnicity**	**207**		**164**		**12**		**16**		**0**		**225**		**150**		**774**	
	Black	43	20.8	6	3.7	2	16.7	2	12.5	0	0.0	14	6.2	13	8.7	80	10.3
	Asian/PI	20	9.7	38	23.2	0	0.0	1	6.3	0	0.0	21	9.3	12	8.0	92	11.9
	Hispanic	37	17.9	25	15.2	1	8.3	7	43.8	0	0.0	59	26.2	31	20.7	160	20.7
	White	90	43.5	76	46.3	8	66.7	6	37.5	0	0.0	112	49.8	80	53.3	372	48.1
	Multiracial/other	5	2.4	2	1.2	0	0.0	0	0.0	0	0.0	2	0.9	0	0.0	9	1.2
	Not reported	12	5.8	17	10.4	1	8.3	0	0.0	0	0.0	17	7.6	14	9.3	61	7.9
IARC	**Infection outcome**	**81**		**76**		**4**		**3**		**7**		**49**		**88**		**309**	
	Non‐cancer	45	55.6	28	36.8	2	50.0	0	0.0	3	42.9	28	57.1	37	42.0	144	46.6
	Cervical cancer	36	44.4	48	63.2	2	50.0	3	100.0	4	57.1	21	42.9	51	58.0	165	53.4
	**Geographic Region**	**81**		**76**		**4**		**3**		**7**		**49**		**88**		**309**	
	East Asia	1	1.2	37	48.7	0	0.0	0	0.0	0	0.0	3	6.1	17	19.3	58	18.8
	Europe	1	1.2	0	0.0	0	0.0	0	0.0	0	0.0	24	49.0	9	10.2	34	11.0
	North Africa	1	1.2	4	5.3	0	0.0	0	0.0	0	0.0	2	4.1	6	6.8	13	4.2
	North America	0	0.0	2	2.6	0	0.0	0	0.0	0	0.0	2	4.1	2	2.3	6	1.9
	Oceania	0	0.0	6	7.9	0	0.0	0	0.0	0	0.0	3	6.1	0	0.0	9	2.9
	South Asia	4	4.9	14	18.4	0	0.0	0	0.0	0	0.0	8	16.3	7	8.0	33	10.7
	S/C America	3	3.7	10	13.2	0	0.0	2	66.7	0	0.0	5	10.2	11	12.5	31	10.0
	SS Africa	71	87.7	3	3.9	4	100.0	1	33.3	7	100.0	2	4.1	36	40.9	125	40.5

*Note*: A3 excluded from this table due to counts ≤1.

Abbreviations: ADC, adenocarcinoma; AIS, adenocarcinoma in‐situ; Asian/PI, Asian/Pacific Islander; controls, cervical intraepithelial neoplasia (CIN) grade 1 or lower (≤CIN1); CIN2, CIN grade 2; CIN3, CIN grade 3; non‐cancers, includes controls (normal, atypical squamous or glandular cells of undetermined significance [ASCUS], or low‐grade intraepithelial lesion [LSIL] cytology specimens) and HSIL/CIN2/CIN3; SCC, squamous cell carcinoma; S/C America, South/Central America; SS Africa, Sub‐Saharan Africa.

We identified three new sublineages not previously reported, named here as A4, A5, and A6. Each new sublineage differed by 0.5%–1.3% from the other A sublineages and 1.2%–1.4% from the B lineage (Table S5). Although rare, the A6 sublineage was only found in women from Sub‐Saharan Africa (Figure 2). Using a large dataset of HPV45 genomes enabled us to identify which subclades were enriched in certain regions. A1.2 and A1.3 were only identified in women from Sub‐Saharan Africa, while other members of A1 (e.g., A1.1) were much more widespread. A2.2, B1.1, and B2.1 were predominantly found in women from Asian regions, and B1.2 was primarily found in women from Europe (Table S6).

### 3.2 HPV45 sublineage associations with histology‐specific precancer/cancer

Using data from the PaP cohort, we evaluated sublineage associations with precancer/cancer risk compared to the more common sublineage, A1. The A2 (OR = 3.9, 95% CI = 1.9–8.5, *p* = 0.0003), B1 (OR = 2.7, 95% CI = 1.4–5.8, *p* = 0.007), and B2 (OR = 3.3, 95% CI = 1.6–7.3, *p* = 0.002) sublineages were each significantly associated with higher risks of CIN3+ (Table 2). By histology, we observed stronger associations for A2 (OR = 6.9, 95% CI = 1.0–184, *p* = 0.041), A4/A5 (OR = 14.2, 95% CI = 1.1–459, *p* = 0.041), and a suggestive B1 association (OR = 6.2, 95% CI = 1.1–159, *p* = 0.068) with glandular precancer/cancer (i.e., AIS and ADC) compared to squamous precancer/cancer (i.e., CIN3 and SCC) (Table 2). Results for CIN2+ were similar but attenuated compared to CIN3+ (Table S7), and similar or stronger after excluding samples with an HPV16 coinfection and after excluding both HPV16 and/or HPV18 coinfections (AIS/ADC ORs ranged from 11 to 38; Table 2 and Table S7).

**TABLE 2:** HPV45 sublineage associations with precancer and cancer by histology in the PaP cohort.

Sublineage	Controls		CIN3+					CIN3 and SCC					AIS and ADC				
	*n*	col %	*n*	col %	OR	95% CI	*p*	*n*	col %	OR	95% CI	*p*	*n*	col %	OR	95% CI	*p*
All samples	**557**		**94**					**74**					**19**				
A1	163	29.3	11	11.7	ref	‐‐	‐‐	10	13.5	ref	‐‐	‐‐	1	5.3	ref	‐‐	‐‐
A2	106	19.0	28	29.8	3.91	1.9, 8.5	**0.0003**	22	29.7	3.38	1.6, 7.7	**0.002**	5	26.3	6.89	1.0, 184	**0.041**
A4/A5	21	3.8	2	2.1	1.41	0.1, 7.2	0.652	0	0.0	0.36	0.0, 6.4	0.605	2	10.5	14.22	1.1, 459	**0.041**
B1	164	29.4	30	31.9	2.71	1.4, 5.8	**0.007**	23	31.1	2.29	1.1, 5.2	**0.036**	7	36.8	6.19	1.1, 159	*0.068*
B2	103	18.5	23	24.5	3.31	1.6, 7.3	**0.002**	19	25.7	3.01	1.4, 7.0	**0.007**	4	21.1	5.71	0.8, 157	0.081
Without HPV16 co‐infection	**527**		**73**					**56**					**17**				
A1	157	29.8	8	11.0	ref	‐‐	‐‐	8	14.3	ref	‐‐	‐‐	0	0.0	ref	‐‐	‐‐
A2	95	18.0	20	27.4	4.13	1.8, 10.3	**0.001**	15	26.8	3.10	1.3, 8.0	**0.013**	5	29.4	18.14	1.0, 332	*0.008*
A4/A5	20	3.8	2	2.7	1.95	0.2, 10.8	0.333	0	0.0	0.45	0.0, 8.1	0.602	2	11.8	38.41	1.8, 828	**0.015**
B1	159	30.2	22	30.1	2.72	1.2, 6.7	**0.020**	16	28.6	1.97	0.8, 5.0	0.128	6	35.3	12.84	0.7, 230	*0.030*
B2	96	18.2	21	28.8	4.29	1.9, 10.7	**0.001**	17	30.4	3.48	1.5, 8.8	**0.005**	4	23.5	14.69	0.8, 276	*0.022*

*Note*: OR = odds ratio; CI = confidence interval; Controls = cervical intraepithelial neoplasia (CIN) grade 1 or lower (≤CIN1); CIN3+ = CIN grade 3 and cancer. Squamous precancers and cancers included CIN3 and squamous cell carcinoma (SCC); glandular precancers and cancers included adenocarcinoma in‐situ (AIS) and adenocarcinoma (ADC). The reference for each sublineage comparison was the common A1 sublineage. Significant *p*‐values are given in bold; an italicized *p*‐value indicates a suggestive association where either the 95% CI is above or below one or the *p*‐value is significant. If a cell count was zero, ORs and 95% CIs were estimated by adding a small constant value (0.5) to each cell within the table. Estimates based on <10 cases should be interpreted with caution due to small numbers.

HPV45 sublineage associations with CIN3+ differed when stratified by a woman's self‐reported ethnicity (Table 3). White women with an A1 sublineage infection had a significantly lower risk of CIN3+ compared to White women infected with the other sublineages (OR = 0.4, 95% CI = 0.1–0.9, *p* = 0.027). Among Black women, the A1 sublineage was associated with a suggestively lower risk of CIN3+ (OR = 0.2, 95% CI = 0.0–1.2, *p* = 0.050), while the B2 sublineage was associated with a suggestively greater risk of CIN3+ (OR = 6.1, 95% CI = 0.9–42.1, *p* = 0.031). Asian/PI women with an A2 sublineage infection had the highest risk of CIN3+ compared to Asian/PI women infected with the other sublineages (OR = 5.8, 95% CI = 1.3–37.3, *p* = 0.010).

**TABLE 3:** HPV45 sublineage associations with CIN3+ stratified by a woman's self‐reported ethnicity in the PaP cohort.

Ethnicity/sublineage	Controls		CIN3+		OR	95% CI	*p*
	*n*	Row %	*n*	Row %			
White	**251**		**49**				
A1	68	91.9	6	8.1	0.38	0.1, 0.9	**0.027**
A2	46	75.4	15	24.6	1.97	1.0, 3.9	0.051
B1	78	81.3	18	18.8	1.29	0.7, 2.4	0.437
B2	59	85.5	10	14.5	0.84	0.4, 1.7	0.637
Asian/Pacific Islander	**60**		**12**				
A1	17	94.4	1	5.6	0.23	0.0, 1.84	0.272
A2	20	69.0	9	31.0	5.84	1.3, 37.3	**0.010**
B1	17	94.4	1	5.6	0.23	0.0, 1.8	0.272
B2	6	85.7	1	14.3	0.82	0.0, 7.9	>0.999
Hispanic	**110**		**25**				
A1	28	93.3	2	6.7	0.26	0.0, 1.2	0.065
A2	17	81.0	4	19.0	1.04	0.2, 3.7	>0.999
A5	4	66.7	2	33.3	2.29	0.2, 17.1	0.308
B1	42	82.4	9	17.6	0.92	0.4, 2.3	0.839
B2	19	70.4	8	29.6	2.25	0.8, 5.9	0.097
Black	**52**		**8**				
A1	34	94.4	2	5.6	0.18	0.0, 1.2	*0.050*
B1	11	84.6	2	15.4	1.24	0.1, 8.3	>0.999
B2	7	63.6	4	36.4	6.14	0.9, 42.1	*0.031*

*Note*: OR = odds ratio; CI = confidence interval; CIN3+ = cervical intraepithelial neoplasia grade 3 and cancer; controls are CIN1 or lower. The reference group is all other sublineages combined among a specific self‐reported ethnicity group except for the tested sublineage. Significant *p*‐values are bold; an italicized *p*‐value indicates a suggestive association where either the 95% CI is above or below one or the *p*‐value is significant.

### 3.3 Individual viral SNPs associated with glandular lesions

**FIGURE 3:**
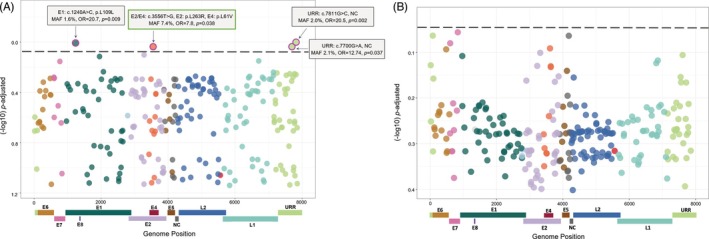
HPV45 genome SNPs associated with (A) glandular or (B) squamous precancer and cancer in the PaP cohort. A total of 262 common SNPs with a minor allele frequency (MAF) ≥1% across the HPV45 genome were evaluated for associations with glandular lesions, and 259 common SNPs for squamous lesions. The *y*‐axis shows the corrected *p*‐values on a logarithm scale for each SNP association. The dashed line illustrates the FDR significance threshold (adjusted *p*‐value). The *x*‐axis illustrates the HPV45 genome positions and viral gene regions by the colored key.

We investigated individual HPV45 SNP associations with CIN2+, CIN3+, and by histologic outcome (AIS/ADC and CIN3/SCC) in the PaP cohort. There was a total of 2123 polymorphic sites (i.e., sites with SNPs) among all samples; 1602 SNPs were rare with an MAF <1% and 521 were common with an MAF ≥1%. Of the 262 common SNPs observed in glandular precancer/cancer (AIS/ADC), four independent SNPs were significantly associated with AIS/ADC: E1 SNP, OR = 20.7, 95% CI = 4.7–91.8; E2/E4 SNP, OR = 7.8, 95% CI = 2.5–24.5; URR SNP, OR = 12.7, 95% CI = 3.0–54.2; URR SNP, OR = 20.5, 95% CI = 5.4–77.6 (Figure 3A). These SNP MAFs ranged from 1.6% to 7.4%; SNP details are summarized in Table S8. There was only one significant SNP that was nonsynonymous. This SNP mapped to the E2/E4 region (T3556G) and was nonsynonymous in both E2 (L263R) and E4 (L61V). None of these four SNPs were lineage‐defining, but the E1 and two URR SNPs were lineage‐specific. That is, they only were detected in women with an A2 sublineage, but not all individuals with A2 had this SNP, so they were not diagnostic. In contrast, there were 11 significant SNPs associated with CIN3+; however, no significant SNPs were associated with CIN2+ or squamous precancer/cancer (CIN3/SCC; Figure 3B) after FDR correction.

To explore the impact of the E2 nonsynonymous SNP at residue 263, we predicted the 3D structure of the HPV45 E2 protein. AlphaFold2^29^ predicted the region with residue 263 as disordered (Figure S1), although with low confidence, with a low pLDDT score (Figure S3). We then predicted how mutations at this residue, L263, would impact the thermodynamic stability of E2 for all 19 amino acid mutations. Interestingly, we observed that the L263R SNP is predicted to make E2 the most thermodynamically stable, with the highest ddG (Table S10).

We further evaluated the combined effect of rare genetic variants by viral gene region and case–control status. There were no significant differences between the cases and controls after FDR correction for multiple tests (Table S9). This differs from our previous study of HPV16 that showed that the controls had more non‐silent rare variants in the E7 gene compared to cases.^21^ There was a suggestive difference between more rare variants in the URR region in CIN2+ cases compared to controls, but this did not remain significant after FDR correction.

## 4 DISCUSSION

We conducted the largest study of HPV45 genome variation to date and uncovered novel sublineages, described a new level of classification [subclades], identified variable risks associated with sublineages and viral SNPs, and analyzed evidence of risk modification by ethnicity. Our study included 1083 HPV45 samples from a large US cohort and worldwide collection sites, which enabled us to evaluate sublineages and individual SNPs associated with precancer and cancer. We show that the A2, B1, and B2 sublineages were associated with an increased risk of all cervical precancer/cancer. The strongest associations were observed for these sublineages, plus A4/A5, which had an increased risk of glandular precancer/cancer. The A2 sublineage was associated with more precancer and cancer in Asian/PI women in the U.S. compared to the other self‐reported ethnicities, and A2 was most prevalent in East Asia compared to other geographic regions. Across the viral genome, four individual viral SNPs were significantly associated with glandular lesions, including a nonsynonymous variant in the overlapping E2/E4 ORFs.

The identification of specific HPV45 sublineages and SNPs strongly linked to glandular lesions could potentially be used to help inform risk stratification for ADC. Current cervical cancer screening in developed countries has not effectively reduced the incidence of ADC, likely due to the difficulty in detecting glandular precancer/cancer that develops deeper in the endocervical canal.^6^, ^31^ ADC accounts for ~10% of cervical cancers globally; however, in developed regions with cervical cancer screening programs, it accounts for ~25% of new cases.^32^, ^33^, ^34^, ^35^, ^36^, ^37^ We identified specific sublineages and SNPs linked to increased risk of AIS/ADC. One of these SNPs was an independent SNP (i.e., not lineage or sublineage defining) that resulted in an amino acid change in E4 (Leu to Val) and E2 (Leu to Arg). The E2 amino acid change replaces a charged amino acid with a hydrophobic one and may disrupt existing electrostatic interactions. Out of all possible amino acid changes at this E2 position (263), a change to Arg is most thermodynamically stable, suggesting that the mutated protein might be more efficacious/stable with this change. Based on our 3D structure predictions of HPV45 E2, this region of E2 is disordered but predicted with low confidence. However, further studies are needed to follow up and evaluate the functional implications of this variant and association with glandular lesions. Atypical glandular cell (AGC) cytology is thought to be the precursor to AIS and ADC and potentially can act as a predictor of risk of glandular precancer and cancer.^3^ In this analysis, a total of 19 women with an AGC cytology had a subsequent histologic diagnosis of CIN2 (5), CIN3 (4), AIS (7), ADC (2), or SCC (1), indicating they likely had a high‐grade lesion.

Our results confirm and expand upon the findings from our previous study that showed that the HPV45 B2 sublineage was associated with more cervical cancer than the non‐B2 sublineages.^8^ Here, using WGS, we were able to identify new sublineages (A4, A5, and A6) in these IARC samples, which had not been identified with previously targeted E6/E7 sequencing, and we further refined the sublineage risk differences detected using a large case–control cohort. In the PaP data, B2 was associated with more precancer/cancer in Black women compared to the other sublineages. Although numbers were small, this is consistent with B2 having the strongest association with cancer in women from Africa.^8^

We show that infection with specific HPV45 sublineages and the risk of precancer/cancer is modified by a woman's ethnicity. The A2 sublineage, and specifically the A2.2 subclade of A2, was more prevalent in Asian women and associated with more precancer/cancer in Asian/PI women compared to all other women infected with these variants. In addition, the A1.2 and A1.3 subclades were only found in women from Sub‐Saharan Africa. Similar to some previous studies of other HPV types,^14^, ^15^ this suggests that HPV45 has co‐evolved with specific populations. An increased prevalence may indicate a greater fitness of the virus in this population and a greater likelihood of progression, possibly due to an advantage in evading the host immune surveillance.^9^ However, because the majority of women in our PaP cohort were White, this viral‐host interaction was not as clear for all HPV45 variants and ethnicities with the same origin. For example, the A1 sublineage was most prevalent in women from sub‐Saharan Africa, but in women who self‐reported as Black, there was a lower precancer/cancer association compared to all other women. While we think subclade classifications offer a finer evaluation of evolutionary groupings that may be important in specific populations, these may be harder to delineate in small studies, and we still recommend evaluating sublineages separately. Large studies in diverse populations are needed to more thoroughly explore this viral‐host interaction and to understand how/if it relates to the host's immune response.

An important strength of our study was utilizing a large number of viral whole‐genome sequences, allowing for a higher degree of discrimination by lineage, sublineage, subclades, and SNPs. Although our study is the largest to date, it is still limited by the rarity of glandular lesions, and estimates based on small numbers may be unreliable and should be followed up in additional studies. An additional limitation was the use of self‐reported ethnicity, which is only a surrogate of genetic ancestry; HPV genome studies that incorporate human genetic ancestry would allow for a more accurate evaluation of viral‐host relationships.

In summary, our study identified HPV45 sublineages and SNPs linked to important precancer/cancer risk differences that vary based on lesion histology and population. Importantly, we identify HPV45 variants specifically associated with the risk of glandular precancer/cancer that warrant follow‐up and could improve our understanding of glandular lesion etiology.

## AUTHOR CONTRIBUTIONS

**Aimee J. Koestler:** Writing – original draft; visualization; writing – review and editing; formal analysis; data curation; investigation. **Chase W. Nelson:** Writing – review and editing; methodology; software; formal analysis; investigation. **Meredith Yeager:** Writing – review and editing; validation; methodology; investigation; data curation. **Zigui Chen:** Writing – review and editing; software; methodology. **Sambit K. Mishra:** Writing – review and editing; validation; methodology; software; data curation. **Laurie Burdett:** Validation; investigation; writing – review and editing. **Michael Dean:** Writing – review and editing; investigation. **Elizabeth Suh‐Burgmann:** Writing – review and editing; resources; data curation. **Thomas Lorey:** Writing – review and editing; resources; data curation. **Gary M. Clifford:** Writing – review and editing; resources; investigation; funding acquisition; data curation. **Nicolas Wentzensen:** Writing – review and editing; resources; funding acquisition. **Philip E. Castle:** Writing – review and editing; resources. **Mark Schiffman:** Writing – review and editing; conceptualization; resources; funding acquisition. **Robert D. Burk:** Conceptualization; writing – review and editing; methodology; investigation. **Lisa Mirabello:** Conceptualization; methodology; formal analysis; writing – review and editing; writing – original draft; supervision; funding acquisition; data curation.

## FUNDING INFORMATION

This study was funded by the intramural research program of the Division of Cancer Epidemiology and Genetics, National Cancer Institute (NCI), NIH. This project has been funded in whole or in part with federal funds from the NCI, NIH (HHSN261200800001E); and the NCI (CA‐238592) and the Albert Einstein Cancer Center (P30CA013330) from the NCI (to R. D. B.). Work at IARC was supported by a grant from the Institut National du Cancer (INCa), France (SHSESP 16‐006).

## CONFLICT OF INTEREST STATEMENT

Where authors are identified as personnel of the International Agency for Research on Cancer/World Health Organization (IARC/WHO), the authors alone are responsible for the views expressed in this article, and they do not necessarily represent the decisions, policy, or views of IARC/WHO. The content of this publication does not necessarily reflect the views or policies of the Department of Health and Human Services, nor does mention of trade names, commercial products, or organizations imply endorsement by the US Government.

## ETHICS STATEMENT

For the PaP cohort, the Kaiser Permanente Northern California institutional review board (IRB) approved the use of the data, and the National Institutes of Health Office of Human Subjects Research deemed our study exempt from IRB review. For all other NCI studies, local and NCI IRBs approved the studies. Women could opt out of having their residual cervical specimens retained in the PaP cohort, which are discarded. For the IARC collection, both local and IARC ethical committees approved our study. Additionally, written and oral consent were obtained.

## Supporting information


Data S1.

Data S2.

## Data Availability

The HPV45 genome sequences used in our analyses are available in GenBank under the accession numbers PV418724 to PV419494. Three samples were not included in GenBank due to software restrictions, and these samples are available upon request. Other variables that support the findings of this study are available from the corresponding author upon reasonable request.
