# Supplementary material for: Whole‐genome sequencing of 1,083 HPV45 cases and controls identifies genetic variants associated with glandular cervical lesions
Source: Int J Cancer. 2025 May 5;157(6):1130–41. doi: 10.1002/ijc.35464 (PMC12280842; doi:10.1002/ijc.35464)

Data Supplement

Title: Whole-genome sequencing of 1,083 HPV45 cases and controls identifies genetic variants associated with glandular cervical lesions

Authors: Aimee J. Koestler, Chase W. Nelson, Meredith Yeager, Zigui Chen, Sambit K. Mishra, Laurie Burdett, Michael Dean, Elizabeth Suh-Burgmann, Thomas Lorey, Gary M. Clifford, Nicolas Wentzensen, Philip E. Castle, Mark Schiffman, Robert D. Burk, Lisa Mirabello

Table of Contents:

Supplemental Table S1 ………………………………………………………….... pages 2–3

Supplemental Table S2 ………………………………………………………….... pages 4–6

Supplemental Table S5 …………………………………………………………… page 7

Supplemental Table S6 …………………………………………………………… page 8

Supplemental Table S9 …………………………………………………………… page 9

Supplemental Table S10 ………………………………………………………….. page 10

Information on Additional Supplementary Tables …………………………………….. page 11

Supplemental Figure S1 ………………………………………………………….. page 12

Supplemental Figure S2 ………………………………………………………….. page 13

Supplemental Figure S3 ………………………………………………………….. page 14

Table S1. Characteristics of HPV45-positive samples from the PaP cohort and IARC study.

Study	Characteristics		*n*	*col %*
**PaP** *N*=774	Infection outcome	Control	557	72.0%
		CIN2	123	15.9%
		CIN3	69	8.9%
		AIS	14	1.8%
		SCC	5	0.6%
		ADC	5	0.6%
		Cancer (unknown histology)	1	0.1%
	Age at enrolment	18-29	105	13.6%
		30-39	339	43.8%
		40-49	181	23.4%
		50-59	111	14.3%
		60+	38	4.9%
	Self-reported ethnicity	Black	80	10.3%
		Asian/Pacific Islander	92	11.9%
		Hispanic	160	20.7%
		White	372	48.1%
		Multiracial/other	9	1.2%
		Not reported	61	7.9%
	HPV type	HPV45 single infection	483	62.4%
		With HPV16 coinfection only	73	9.4%
		With HPV18 coinfection only	23	3.0%
		With HPV16 and 18 coinfection	7	0.9%
		Other coinfection	188	24.3%
**IARC** *N*=309	Infection outcome	Non-cancer	144	46.6%
		Control	135	43.7%
		Precancer	9	2.9%
		ADC	16	5.2%
		SCC	146	47.2%
		Cancer (unknown histology)	3	1.0%
	Age	17-29	81	26.2%
		30-39	79	25.6%
		40-49	85	27.5%
		50-59	40	12.9%
		60+	22	7.1%
		Unknown	2	0.6%
	Geographic region	East Asia	58	18.8%
		Europe	34	11.0%
		North Africa	13	4.2%

		North America	6	1.9%
		Oceania	9	2.9%
		South Asia	33	10.7%
		South/Central America	31	10.0%
		Sub-Saharan Africa	125	40.5%
	HPV type	HPV45 single infection	203	65.7%
		With HPV16 coinfection	13	4.2%
		With HPV18 coinfection	16	5.2%
		With HPV16 and 18 coinfection	1	0.3%
		Other coinfection	76	24.6%

Summary statistics from the PaP and IARC studies. PaP infection outcomes: Controls = ≤CIN1; CIN2 = cervical intraepithelial neoplasia (CIN) grade 2; CIN3 = CIN grade 3; AIS = adenocarcinoma in situ; SCC = squamous cell carcinoma, ADC = adenocarcinoma. IARC Infection outcomes: Non-cancers = controls (normal, atypical squamous or glandular cells of undetermined significance [ASCUS], or low-grade intraepithelial lesion [LSIL] cytology specimens) and HSIL/CIN2/CIN3; ADC = adenocarcinoma, which includes adenosquamous.

Table S2. IARC HPV45-positive sample counts by region, country, status, and sublineage.

Region	Country	Status	Sublineage								Totals
			A1	A2	A3	A4	A5	A6	B1	B2	*n*
East Asia	China	Control		1							1
		HSIL/CIN2/3								2	2
	Indonesia	Cervical Cancer		1						3	4
	Korea	Control								1	1
	Mongolia	Control		9					1	1	11
	Philippines	Control		1							1
		Cervical Cancer	1	25					2	5	33
	Thailand	Cervical Cancer								5	5
Europe	Georgia	Control							9	2	11
		HSIL/CIN2/3								1	1
		Cervical Cancer							5	4	9
	Poland	Control							7		7
		Cervical Cancer							3	1	4
	Spain	Cervical Cancer	1							1	2
North Africa	Algeria	Control		1							1
		Cervical Cancer		2					1	3	6
	Morocco	Control	1								1
		Cervical Cancer		1					1	3	5
North America	Canada	Cervical Cancer		2					1		3
	Cuba	Cervical Cancer							1	1	2
	USA	Cervical Cancer								1	1
Oceania	Fiji	Control		6					3		9
South Asia	Bhutan	Control	2	5					5	3	15
		HSIL/CIN2/3								1	1
		Cervical Cancer	1	1						1	3

	India	Control		3					1		4
		HSIL/CIN2/3		1							1
		Cervical Cancer		3					2		5
	Iran	Control	1	1						1	3
	Nepal	Control								1	1
**South/Central America**	Argentina	Control							1		1
		Cervical Cancer							1	1	2
	Bolivia	Cervical Cancer		3						1	4
	Brazil	Cervical Cancer	1							5	6
	Chile	Cervical Cancer		1					1	2	4
	Panama	Cervical Cancer		3			1		1	1	6
	Paraguay	Cervical Cancer	2				1		1	1	5
	Peru	Cervical Cancer		3							3
**Sub-Saharan Africa**	Guinea	Control	10								10
		HSIL/CIN2/3	1								1
		Cervical Cancer	5								5
	Kenya	Cervical Cancer	8	3						7	18
	Mali	Cervical Cancer	6				1			2	9
	Nigeria	Control	10		1					2	13
	Rwanda	Control	19			2		3	1	20	45
		HSIL/CIN2/3								2	2
	South Africa	HSIL/CIN2/3	1								1
		Cervical Cancer	8			2		2	1	3	16
	Tanzania	Cervical Cancer	2					1			3

	Uganda	Cervical Cancer	1					1			2
**Totals**			81	76	1	4	3	7	49	88	309

Cervical cancer includes cervical intraepithelial neoplasia grade 3 and cancer (CIN3+), squamous cell carcinoma (SCC), adenocarcinoma (ADC), and invasive cervical cancer. Non-cancers = controls (normal, atypical squamous or glandular cells of undetermined significance [ASCUS], or low-grade intraepithelial lesion [LSIL] cytology specimens) and HSIL/CIN2/CIN3.

Table S5. Pairwise distances of HPV45 sublineage references.

	A1|EF202163	A2|EF202159	A3|KC470256	A4|PAP229916	A5|PAP282534	A6|IRC203559	B1|EF202162	B2|KC470259
**A1|EF202163**								
**A2|EF202159**	0.61%							
**A3|KC470256**	0.52%	0.77%						
**A4|PAP229916**	1.08%	1.25%	0.91%					
**A5|PAP282534**	0.99%	1.17%	0.93%	0.57%				
**A6|IRC203559**	0.50%	0.59%	0.60%	1.06%	1.00%			
**B1|EF202162**	1.20%	1.38%	1.24%	1.39%	1.29%	1.16%		
**B2|KC470259**	1.21%	1.35%	1.25%	1.39%	1.29%	1.19%	0.79%	

A4, A5, and A6 (in yellow highlight) are new sublineages identified in this study. A4 and A5 were sublineages identified in the PaP cohort and A6 was identified in the IARC study.

Table S6. HPV45 subclade associations with all cancers stratified by geographic region in the IARC study.

	Total	Controls		All Cancers				
		n	row %	n	row %	OR	95% CI	*p*
**Region**								
East Asia	**49**	**15**		**34**				
A1.1	1	0	0.0%	1	100.0%	--	--	--
A2.1	2	0	0.0%	2	100.0%	--	--	--
A2.2	25	2	8.0%	23	92.0%	15.97	2.72, 177	**2.650E-04**
A2.3	10	9	90.0%	1	10.0%	0.02	4.17E-04, 0.20	**1.977E-05**
B1.2	1	1	100.0%	0	0.0%	--	--	--
B2.1	10	3	30.0%	7	70.0%	1.07	0.19, 7.61	>0.999
Europe	**15**	**10**		**5**				
B1.2	15	10	66.7%	5	33.3%	--	--	--
North Africa	**4**	**1**		**3**				
A2.1	1	0	0.0%	1	100.0%	--	--	--
A2.3	3	1	33.3%	2	66.7%	--	--	--
North America	**2**	**0**		**2**				
A2.3	2	0	0.0%	2	100.0%	--	--	--
Oceania	**6**	**6**		**0**				
A2.1	1	1	100.0%	0	0.0%	--	--	--
A2.3	5	5	100.0%	0	0.0%	--	--	--
South Asia	**28**	**21**		**7**				
A1.1	3	3	100.0%	0	0.0%	--	--	--
A2.1	2	0	0.0%	2	100.0%	--	--	--
A2.2	7	6	85.7%	1	14.3%	0.51	0.01, 6.95	>0.999
A2.3	5	4	80.0%	1	20.0%	0.88	0.01, 13.30	>0.999
B1.1	8	6	75.0%	2	25.0%	1.32	0.09, 15.16	>0.999
B2.1	3	2	66.7%	1	33.3%	1.93	0.03, 46.96	0.539
South/Central America	**11**	**0**		**11**				
A2.1	1	0	0.0%	1	100.0%	--	--	--
A2.3	9	0	0.0%	9	100.0%	--	--	--
B1.2	1	0	0.0%	1	100.0%	--	--	--
Sub-Saharan Africa	**34**	**16**		**18**				
A1.1	4	1	25.0%	3	75.0%	2.37	0.16, 143	0.615
A1.2	18	8	44.4%	10	55.6%	0.64	0.05, 5.89	>0.999
A1.3	6	6	100.0%	0	0.0%	--	--	--
A2.2	2	0	0.0%	2	100.0%	--	--	--
A2.3	1	0	0.0%	1	100.0%	--	--	--
B1.1	1	0	0.0%	1	100.0%	--	--	--
B2.1	2	1	50.0%	1	50.0%	0.70	0.01, 60.29	>0.999

OR = odds ratio, CI = confidence interval. All cancers include invasive cervical cancer, squamous cell carcinoma (SCC), and adenocarcinoma (ADC). Controls include normal, atypical squamous or glandular cells of undetermined significance [ASCUS], or low-grade intraepithelial lesion [LSIL] cytology specimens; HSIL/CIN2/CIN3 (n = 9) were not included in this analysis. The reference is "all others," which only includes samples which had a non-zero count for both cases and controls. Fisher's exact test was used when counts in at least one cell in each contingency table was less than five. Sublineages with a zero value for a single outcome were excluded from OR, 95%CI, and *p*-value calculations (significant *p*-values are bolded).

Table S9. Rare variant burden analysis for all HPV45 sublineages in the PaP cohort.

Viral Gene/Region	Controls, *N*=557		CIN2+, *N*=217				CIN3+, *N*=94			
	n	% of controls	n	% of cases	*p*	*p*-FDR	n	% of cases	*p*	*p*-FDR
**URR**	110	19.7%	55	25.3%	**0.018**	0.180	20	21.3%	0.303	0.904
**E6**	22	3.9%	5	2.3%	0.630	0.966	1	1.1%	0.231	0.904
**E7**	16	2.9%	6	2.8%	0.714	0.966	2	2.1%	>0.999	0.999
**E1**	66	11.8%	26	12.0%	0.944	0.966	10	10.6%	0.838	0.931
**E2**	60	10.8%	25	11.5%	0.909	0.966	13	13.8%	0.498	0.904
**E4**	19	3.4%	8	3.7%	0.636	0.966	4	4.3%	0.546	0.904
**E5**	17	3.1%	13	6.0%	0.054	0.270	4	4.3%	0.517	0.904
**E8**	0	0.0%	1	0.5%	0.269	0.897	1	1.1%	0.139	0.904
**L1**	64	11.5%	20	9.2%	0.966	0.966	7	7.4%	0.633	0.904
**L2**	130	23.3%	51	23.5%	0.617	0.966	22	23.4%	0.814	0.931
**Total**	325	58.3%	131	60.4%			54	57.4%		

Controls = cervical intraepithelial neoplasia (CIN) grade 1 or lower (≤CIN1); CIN3+ = CIN grade 3 and cancer; L1 = late gene 1; L2 = late gene 2; E1 = early gene 1; E2 = early gene 2; E4 = early gene 4; E5 = early gene 5; E6 = early gene 6; E7 = early gene 7; URR = upstream regulatory region. *p*-value = Fisher's exact test when counts less than five; when counts are greater than five, General Linear model used. Significant *p*-values are bolded.

Table S10. Mutation sensitivity analysis for the HPV45 E2 protein, residue L263.

Mutation	Predicted ddG	Effect on protein stability
L263R	0.52	Stabilizing
L263K	0.34	Stabilizing
L263H	0.23	Stabilizing
L263G	0.09	Stabilizing
L263S	0.03	Stabilizing
L263E	-0.09	Destabilizing
L263N	-0.09	Destabilizing
L263P	-0.12	Destabilizing
L263M	-0.18	Destabilizing
L263V	-0.2	Destabilizing
L263D	-0.24	Destabilizing
L263Y	-0.28	Destabilizing
L263C	-0.31	Destabilizing
L263I	-0.32	Destabilizing
L263F	-0.4	Destabilizing
L263Q	-0.46	Destabilizing
L263W	-0.48	Destabilizing
L263T	-0.5	Destabilizing
L263A	-1	Destabilizing

Mutation: L263 was mutated into one of these 19 amino acids. Predicted ddG: The thermodynamic stability change predicted by DynaMut2; positive values indicate increased stability upon mutation, and negative values indicate decreased stability.

Information on Additional Supplementary Tables

Tables S3, S4, S7, and **S8** can be found in the additional supplementary materials entitled "Supplementary_Tables_HPV45.xlsx".

- **Table S3.** Summary of sequencing coverage and quality statistics of the HPV45 genome sequencing data.

- **Table S4.** Characteristics of each HPV45 sublineage in 774 samples from the PaP cohort and 309 samples from the IARC study.

- **Table S7.** HPV45 sublineage associations with precancer and cancer by histology in the PaP cohort.

- **Table S8.** Associations between individual HPV45 SNPs and AIS/ADC in the PaP cohort.

Fig S1. HPV45 sublineage distribution by geographic regions using 165 cancer samples collected by IARC from 26 countries. The light blue highlighted countries represent the sites where cancer samples were collected. The sublineage color legend identifies the corresponding color for each sublineage to illustrate the sublineage distribution in each pie chart for the main geographic regions. The distribution of sublineages in the NCI-KPNC PaP cohort is shown as an inset pie chart to display the distribution in the U.S. This figure is a more detailed version of Fig 2 describing the distribution of cancer samples.

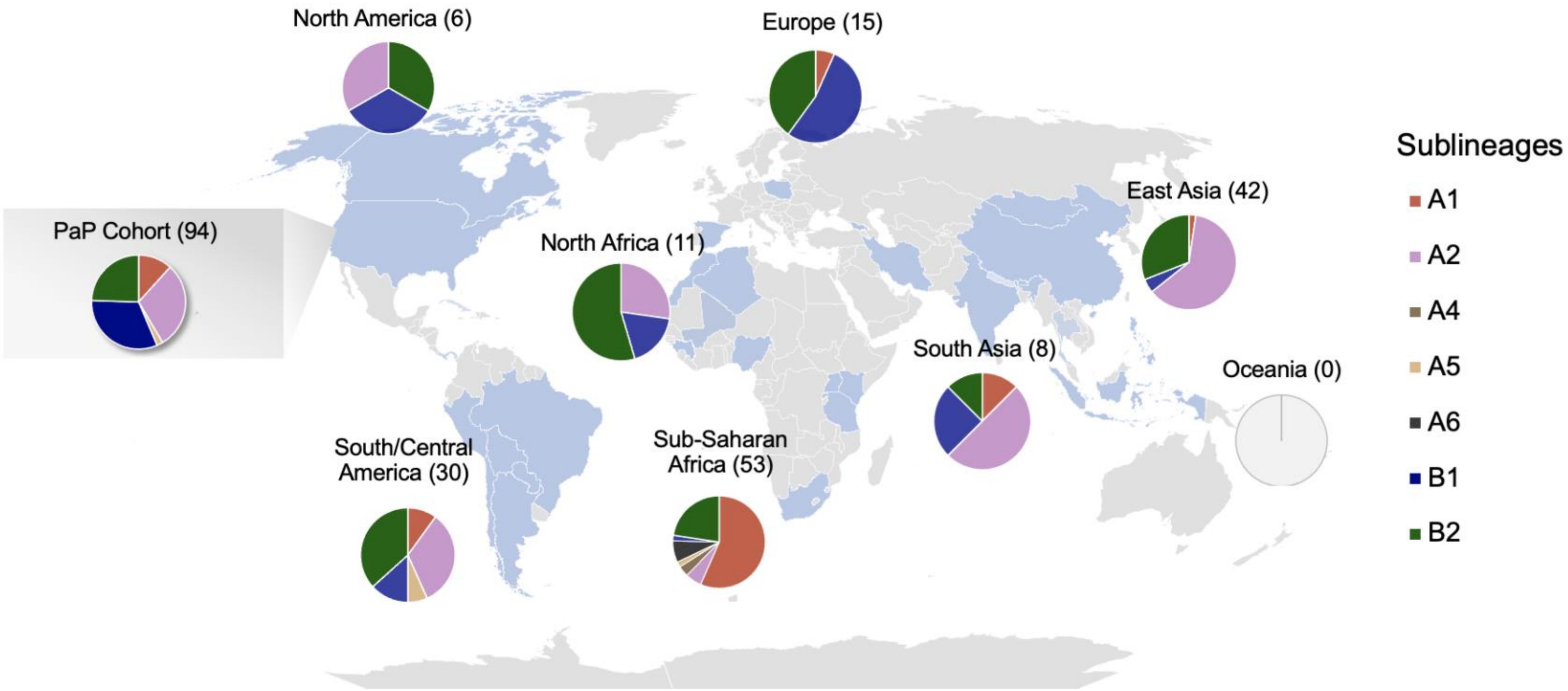

Fig S2. 3D structure of HPV45 E2 protein. The 3D structure of the HPV45 E2 protein predicted by AlphaFold2 is shown. The N-terminal domain (left) and the C-terminal domain (right) are connected by the intrinsically disordered region of the protein (residues 194-290, shown in purple) as predicted by AlphaFold2.

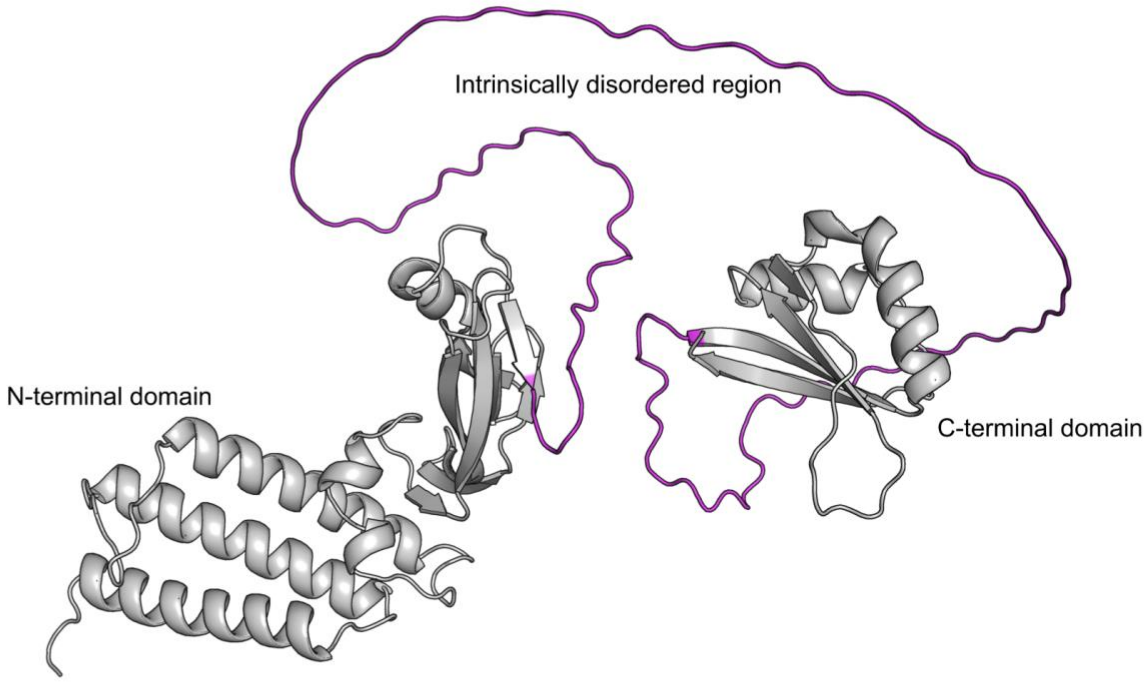

Fig S3. Predicted Local Distance Difference Test (pLDDT) scores for the HPV45 E2 protein. The pLDDT score for each residue in the predicted HPV45 E2 structure is reported by AlphaFold2. Residues with high pLDDT scores (blue) have high confidence, whereas residues with low pLDDT (orange/red) scores have low confidence.

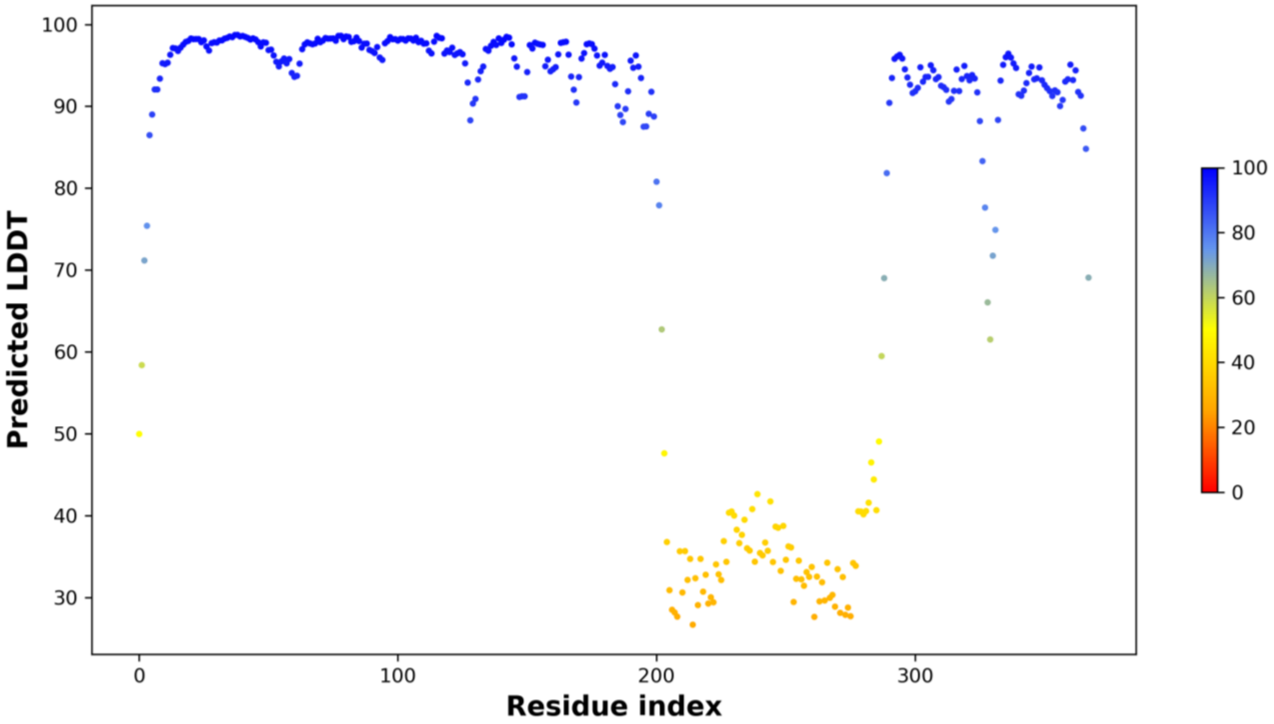

Supplement: Supplementary file 1 — Data S1. [file IJC-157-1130-s001.pdf]
